# Task syndromes: linking personality and task allocation in social animal groups

**DOI:** 10.1093/beheco/araa083

**Published:** 2021-02-06

**Authors:** J C Loftus, A A Perez, A Sih

**Affiliations:** 1 Department of Anthropology, University of California at Davis, Davis, CA, USA; 2 Department for the Ecology of Animal Societies, Max Planck Institute of Animal Behavior, Radolfzell, Germany; 3 Department of Biology, University of Konstanz, Konstanz, Germany; 4 Centre for the Advanced Study of Collective Behaviour, University of Konstanz, Konstanz, Germany; 5 Department of Entomology, University of California at Davis, Davis, CA, USA; 6 Department of Environmental Science and Policy, University of California at Davis, Davis, CA, USA

**Keywords:** division of labor, personality, social behavior, task allocation

## Abstract

Studies of eusocial insects have extensively investigated two components of task allocation: how individuals distribute themselves among different tasks in a colony and how the distribution of labor changes to meet fluctuating task demand. While discrete age- and morphologically-based task allocation systems explain much of the social order in these colonies, the basis for task allocation in non-eusocial organisms and within eusocial castes remains unknown. Building from recent advances in the study of among-individual variation in behavior (i.e., animal personalities), we explore a potential mechanism by which individuality in behaviors unrelated to tasks can guide the developmental trajectories that lead to task specialization. We refer to the task-based behavioral syndrome that results from the correlation between the antecedent behavioral tendencies and task participation as a task syndrome. In this review, we present a framework that integrates concepts from a long history of task allocation research in eusocial organisms with recent findings from animal personality research to elucidate how task syndromes and resulting task allocation might manifest in animal groups. By drawing upon an extensive and diverse literature to evaluate the hypothesized framework, this review identifies future areas for study at the intersection of social behavior and animal personality.

## Teaser

Consistent individual differences in seemingly task-independent behavioral tendencies can potentially drive task allocation in animal groups. Recent studies have found that animal groups containing behaviorally diverse individuals can outperform less diverse groups. In reviewing the literature, we find support for the hypothesis that efficient task allocation driven by variation among groupmates in task-independent behavior contributes to the success of diverse groups, and we generate predictions for future exploration of this phenomenon.

## INTRODUCTION

Task allocation, the process by which groups distribute individuals among tasks to meet variable task demand (Gordon 1996), may be a key adaptation driving the success of large, ecologically dominant societies (e.g., ant societies; Oster and Wilson 1978). In eusocial insects, which have been the focus of task allocation research, age- and morphologically-based caste systems often determine broad patterns of task specialization (Oster and Wilson 1978; Seeley 1982). However, task allocation cannot be completely explained by variation in morphology and age alone. For instance, some eusocial insects demonstrate task allocation without any apparent worker caste system (Gordon 2016), and conspicuous task allocation patterns have been observed in non-eusocial systems as well (*Dictyosteliida (Amoebozoa)*: Sathe et al. 2010; *Pseudoscorpionida*: Tizo-Pedroso and Del-Claro 2011; *Lepidoptera*: Underwood and Shapiro 1999, *Rodentia*: Hurtado et al. 2013; *Cetartiodactyla*: Gazda et al. 2005, Mastick 2016; *Carnivora*: Stander 1992; *Passeriformes*: Arnold et al. 2005; *Cichliformes*: Bruintjes and Taborsky 2011; *Primates*: Boesch 2002). A gap, therefore, exists in our understanding of how early forms of task allocation manifest and are regulated in social systems. We propose that, under certain circumstances, task allocation might arise when variation among individuals in behavioral tendencies unrelated to major tasks becomes reinforced and elaborated in such a way that causes individuals to specialize on different tasks. We refer to the resulting correlation between antecedent behavioral tendencies and later task participation as a task syndrome, and we suggest that these task syndromes have the potential to occur across a variety of social systems.

Animal personality, or the component of behavioral variation in a population that is explained by among-individual variation (Dingemanse et al. 2010), has important ecological and evolutionary consequences (Sih et al. 2004; Bell 2007; Réale et al. 2007; Sih et al. 2012). An individual’s fitness can depend critically upon how well its central behavioral tendencies (i.e., behavioral types) are suited to the particular environment that it experiences (Dall et al. 2004; Réale et al. 2010). For social animals, groupmates represent a significant component of the environment that an individual experiences, and accordingly, personality can strongly impact how social animals affect and are affected by their groupmates (Webster and Ward 2011). Among-individual behavioral variation has consequences not only for individuals, but for entire collectives as well. A group’s composition of behavioral types can affect its collective behavior and performance (Sih 2013; Farine et al. 2015; Montiglio et al. 2017; Jolles et al. 2017). In aggregate, recent research demonstrates that, across several taxa and across several contexts, groups containing greater among-individual behavioral variation can outperform homogeneous groups (Table 2). The mechanism generating these observed disparities in group performance remains unclear. Accordingly, we aim to:

1. Present a conceptual framework connecting social interactions to the reinforcement of among-individual behavioral variation within groups and a group’s adaptive task allocation system;
2. Thoroughly review the evidence evaluating each hypothesized component of the framework;
3. Critically appraise the framework by suggesting circumstances under which it may or may not apply and presenting alternatives to those hypotheses set forth in the framework components;
4. Establish predictions that can guide future tests of the robustness and generalizability of the proposed framework, and suggest a general method for identifying task syndromes.

**Figure 1:**
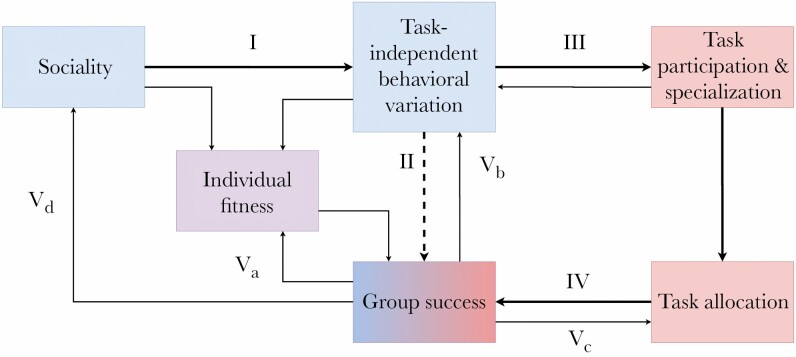
Framework outlining conceptual connections and feedback loops reviewed in this manuscript. Numbered arrows correspond to numbered sections in the main text. Previous research has focused on the connections between sociality, among-individual variation in task-independent behavioral tendencies and group success, and those between task specialization/proficiency, task allocation, and group success. We urge future research to investigate how among-individual variation in task-independent behaviors can lead to group success, and specifically examine if it is through functionally advantageous task allocation. The overarching feedback loop that connects all of these concepts has yet to be fully studied in any social system. We suggest that among-individual variation in task-independent behavioral types and subsequent task syndromes provide a pathway that connects sociality to task allocation.

Our conceptual framework (Figure 1) consists of several hypothesized links that connect the formation of social groups to the improved group performance through task allocation. In the first link, we propose that increased social interactions that result from group formation lead to greater among-individual variation in behaviors that are independent of tasks (Figure 1; I). Secondly, we hypothesize that this among-individual variation improves group performance (Figure 1; II). To explain how this variation improves performance, we suggest that among-individual variation in task-independent behaviors may guide task participation and thus specialization (Figure 1; III) and that the task allocation regime resulting from this specialization enhances group performance (Figure 1; IV). Lastly, we hypothesize that a group’s performance affects several upstream components of the framework (Figure 1; V), thus initiating feedbacks that further elaborate and hone preliminary forms of task allocation.

## CLARIFYING TERMINOLOGY AND THE CONCEPTS OF PERSONALITY AND PLASTICITY

Personality is the proportion of behavioral variation in a population that is explained by the variation among individuals (Dingemanse et al. 2010). Broadly speaking, it implies among-individual variation at the population-level and relatively consistent behavior at the individual-level (Sih et al. 2004; Sih and Bell 2008). While personality is a population-level concept, an individual’s behavioral type is described by its mean behavior relative to the population mean in a given axis of variation.

According to these definitions, task specialization, which refers to consistent individual differences in task-related behaviors such as brood care or nest defense, represents personality as well. Thus, an individual can be described by its behavioral type along several axes of variation, some clearly task-related and others that show no explicit connection to task performance. We refer to the latter behaviors as task-independent behaviors, and they are behaviors that: 1) are not explicitly involved in carrying out a task and 2) can be observed and measured when individuals are not participating in a task or are experimentally deprived of the opportunity to participate in a task. At the population-level, any quantifiable association between two axes of behavioral variation, whether they are task-independent or task-related, can be described as a behavioral syndrome (Sih et al. 2004). Here, we focus on the notion that among-individual variation in task-independent behaviors could be elaborated and reinforced in such a way that leads to among-individual variation in individual task participation, resulting in a task syndrome.

Among-individual variation in behavior, or personality, is not mutually exclusive from within-individual variation, or plasticity. Together, these two components define the total variation within a population, and their contribution to the total variation is illustrated well by the behavioral reaction norm approach (Dingemanse et al. 2010). An individual’s reaction norm represents its behavior as a function of an environmental gradient in a given period of time. The slope of the individual’s reaction norm represents the within-individual component of variation in the given time frame, indicating how much the individual’s behavior will change in response to a change in the environment. The differences between individuals’ behavior within a given environmental context, or differences that persist across several environmental contexts represents the among-individual component of behavioral variation, or personality.

Importantly, plasticity can occur at two scales. Activational plasticity reflects the variation in behavior that an individual exhibits across environmental contexts at a given time (Snell-Rood 2013). Synonymous with the within-individual component of variation reflected in behavioral reaction norms, activational plasticity is represented by movement along an individual’s behavioral reaction norm as the individual transitions between environmental contexts. However, there is also a time-depth component to plasticity not recognizable in a reaction norm from a single time period. Developmental plasticity results from the same genotype expressing different phenotypes in different environments as a result of the environments favoring divergent developmental trajectories (Stamps and Groothuis 2010; Snell-Rood 2013; Stamps 2016). A critical difference here is that there is a time-lag between experiencing a particular environment and exhibiting a behavioral change, and that this change is typically long-lasting (Stamps 2016). This process is akin to an individual’s entire reaction norm, and thus its behavioral type, gradually shifting over time. Developmental plasticity and genotypic variation together explain personality, through their contribution to behavioral variation among individuals.

Finally, while personality is defined by the proportion of behavioral variation in a population that is among individuals, when a population comprises social animals, the amount of among-individual variation within a social group can vary among groups. That is, just as groups can differ from each other in their genetic diversity, they can differ in behavioral type diversity. We discuss the feedbacks that can affect the amount of behavioral variation among individuals in social groups, and how this variation affects task allocation and group performance.

## SOCIAL LIVING CAN DIRECTLY LEAD TO AN INCREASE IN AMONG-INDIVIDUAL BEHAVIORAL VARIATION (**FIGURE 1**; I)

An individual’s behavioral type is reflected in the food that it eats (Toscano et al. 2016), the methods by which it accesses resources (Kurvers et al. 2009b; Carter et al. 2014, 2016) and mating opportunities (Kralj-Fišer et al. 2013), and the anti-predator strategies that it employs (Jones and Godin 2010). Thus, the ecological niche that an individual fills is determined not only by its species-specific behavioral tendencies, but also by its individual-specific behavioral tendencies (Sih et al. 2012). Social animals typically compete directly with their groupmates over access to food, mates, and refuge (Bergmüller and Taborsky 2007). This environment of competition favors niche differentiation (Gause 1934), with a fitness advantage conferred upon those using rare or new strategies to survive and acquire resources and mates. As an individual increasingly employs an advantageous, rare behavioral strategy, its behavioral type shifts away from that of its groupmates. This process known as “social niche specialization” can ensue across the members of the group (Bergmüller and Taborsky 2010). As a result, sociality can drive behavioral variation among individuals via developmental plasticity in a negative-frequency-dependent manner (Wolf et al. 2008; Bergmüller and Taborsky 2010; Sih et al. 2015). Furthermore, as individuals diverge in their behavioral types, positive feedback mechanisms that reduce the possible costs of activational plasticity (for costs of plasticity see Hutcheon et al. 2002; Relyea 2002; Changizi 2003; Niven et al. 2007; Snell-Rood 2012; Snell-Rood 2013) can generate further among-individual variation and within-individual consistency (Wolf et al. 2008; Sih et al. 2015).

Alternatively, a behaviorally diverse group that contains individuals with minimal niche overlap could, in theory, result from individuals that have a similar mean behavior, but high within-individual variation in behavior, such that they often exhibit different behaviors and thus fill different ecological niches at any given time. Individuals in this scenario would have higher activational plasticity but similar behavioral types (i.e., little among-individual variation). This alternative, however, is unlikely because there can be substantial costs and limitations to activational plasticity, and predictability can be beneficial in social situations (Johnstone 2001; Dall et al. 2004; Sih et al. 2004; Johnstone and Manica 2011). If behavioral variation is beneficial, but individuals are highly plastic in their behavior, groups of finite size will occasionally exhibit suboptimal mixtures of behaviors at any given time simply due to stochasticity. The probability of these suboptimal mixtures occurring decreases as the differences between individuals in their mean behavior and the consistency of individual behavior increase.

Indeed, there is strong theoretical support for sociality as a potential driver of among-individual variation in behavior. Agent-based models show that group-living can cause among-individual behavioral variation to arise and subsequently increase in a social group even when groupmates are initially identical (Hemelrijk and Wantia 2005; Oosten et al. 2010). Further work using game theoretical models suggest that the presence of a small number of individuals in a group that can adjust their behavior according to the behavior of others (i.e., “socially aware” individuals) is sufficient to substantially enhance initially small behavioral variations among groupmates (McNamara et al. 2009; Wolf et al. 2011). Empirical support for the ability of individuals within groups to adjust their behavior according to that of their groupmates is widespread (Magnhagen and Staffan 2005; Dyer et al. 2009a), particularly in research on indirect genetic effects, which explicitly considers the effects of neighboring conspecifics’ genotypes on the phenotype of a focal individual (Santostefano et al. 2016; Santostefano et al. 2017; reviewed in Montiglio et al. 2013; Dingemanse and Araya-Ajoy 2015). Thus, group-living can theoretically both create and accentuate initial behavioral variation among groupmates.

Empirical evidence that social interactions can lead to greater among-individual variation in behavior is often consistent with theoretical predictions. Jäger et al. (2019) showed that male crickets that had previously interacted with conspecifics exhibited higher repeatability in a test for aggressive behavior compared with those that had not previously interacted with conspecifics. A common garden study of Eurasian perch, *Perca fluviatilis*, shows that individuals differ consistently in willingness to forage near a predator over time (Hellström and Magnhagen 2011), likely because of social interactions such as facilitation and competition (Magnhagen and Staffan 2005; Oosten et al. 2010). Additionally, formation of groups composed of non-aggressive water striders lead to an increase in among-individual variation, with some individuals becoming hyper-aggressive (Sih and Watters 2005). Early social experiences cause initial behavioral variation that becomes canalized into pronounced among-individual variation in adulthood in several taxa of birds and mammals as well (Plomin and Daniels 1987; Bends and Henkelmann 1998; Groothuis and Carere 2005). Because individuals in social species potentially experience more competition over local resources than those in solitary species, and thus a greater benefit of niche differentiation, social species should exhibit greater among-individual variation than closely related solitary species. Preliminary comparative studies appear to corroborate this expectation (Pruitt et al. 2012; von Merten et al. 2017).

To expand on the research that has grounded this portion of our framework, we suggest further work test the predictions of the hypothesis that social interactions and competition with groupmates leads to an increase in among-individual variation. We would predict, for example, that individuals exposed to more social interactions early in life would exhibit more extreme behavioral types than individuals with fewer social experiences, and that among-individual variation would be greater in groups in resource-poor environments than those in resource-rich environments (Table 1). We would also predict greater among-individual variation in stable social groups than in less stable social aggregations, as there may be little potential for individuals to respond to behavioral types of groupmates with a shift in their own behavioral type when group membership is highly dynamic, especially considering that the developmental plasticity needed for this shift in behavioral type typically results in slow, gradual changes. Further research could also explore circumstances under which this hypothesis breaks down. We might not see social-living lead to an increase in among-individual variation when behavioral conformity is imperative. For example, we might not expect among-individual variation in the willingness to forage under the risk of predation if predation pressure is uniformly high in a particular environment, or if behavioral uniqueness increases the probability of predation (i.e., the oddity effect; Landeau and Terborgh 1986; Parrish et al. 1989)

**Table 1:** Hypotheses and predictions for the effect of social grouping on among-individual behavioral variation

Hypothesis	Predictions	Alternative Hypotheses	Predictions
H 1. Among-individual variation increases after social grouping due to competition with groupmates over limited resources	P 1. Among-individual variation in behavior will be greater after group formation than before group formation P 2. Among-individual variation will increase with a) duration since group formation, b) frequency of social interactions, and c) stability of group membership P 3. Among-individual variation will decrease with an increase in the availability of resources P 4. Among-individual behavioral variation will be greater at the population-level in social species compared with that of solitary species	AH 1. Among-individual variation is unaffected by social interactions AH 2. Among-individual behavioral variation decreases after group formation due to: (see following sub-hypotheses)	P 1. Repeatability of a behavior after group formation will not be significantly different from before group formation P 1. Total variance of a behavioral measure taken after group formation will be lower than the total variance before group formation P 2. The repeatability of the behavior will be lower after group formation than before group formation
		AH 2.1. - benefits of social conformity for predation avoidance	P 3. (continued from above general predictions) Repeatability of behavior will decrease more after group formation in a dangerous environment in comparison to a safe environment
		AH 2.2. - benefits of social conformity for information transfer	P 3. Repeatability of behavior will decrease more after group formation in a highly variable or complex environment in comparison to a consistent or simple environment
		AH 2.3. - the effect of a dominant individual that homogenizes subordinate behavior	P 3. Repeatability of behavior will decrease more in groups where a clear dominant individual emerges
		AH 3. Within-individual variation, but not necessarily among-individual variation, increases after social grouping as a result of competition over limited resources	P 1. Total variance of a behavioral measure taken after group formation will be higher than the total variance before group formation P 2. The repeatability of the behavior will be lower after group formation than before group formation

## VARIATION AMONG GROUP MEMBERS IN TASK-INDEPENDENT BEHAVIORS CAN CORRELATE POSITIVELY WITH GROUP SUCCESS (FIGURE 1; II)

Recent animal personality research has highlighted the importance of group behavioral type composition on group performance (Sih 2013; Farine et al. 2015). Taken together, studies demonstrate that, across several systems and across several contexts, within-group among-individual variation in behavior not explicitly associated with tasks can correlate positively with group success (Table 2). The “social heterosis hypothesis” predicts this pattern, positing that variation among group members should improve, rather than impede, group performance (Nonacs and Kapheim 2007). Furthermore, social heterosis might partially contribute to the heightened success of larger groups, as larger groups are statistically more likely to contain greater among-individual variation and less behavioral conformity than smaller groups (Hellström et al. 2011). In this section, we review studies that show a positive correlation between among-individual variation and group success, evaluate alternative hypotheses that could contribute to this result (Table 3), and put forth a tractable hypothesis for a potentially widespread mechanism that might drive this trend across taxa.

**Table 2:** Summary of evidence for the effect of among-individual variation in task-independent behavior on group performance

Measure of Success	Animal	Description	Reference
Reproductive success	Ant, *Temnothorax longispinosus*	Within-colony variation in exploration lead to an increase in total weight of pupae reared	Modlmeier et al. 2012
	*T. longispinosus* *Parsus major*	Within-colony variation in aggression, but not exploration, predicts total weight of pupae for the colony	Modlmeier and Foitzik 2011
	Social spider, *Anelosimus studiosus*	Mixed groups of aggressive and docile individuals have higher masses of egg cases than homogeneous groups	Pruitt and Riechert 2011
Foraging success	*P. major*	Variation in exploratory behavior allows groups to optimally utilize foraging sites while also maintaining group cohesion	Aplin et al. 2014
	Honey bee, *Apis mellifera*	Individual differences in speed-accuracy strategies during foraging decreases variation in food acquisition	Burns and Dyer 2008
	Three-spined stickleback, *Gasterosteus aculeatus*	Shoals with both bold and shy individuals feed more than shoals of all-bold or all-shy individuals	Dyer et al. 2009a
	Social spider, *Stegodyphus dumicola*	Groups with a bold keystone individual gain 200–300% more weight and had 40% lower mortality than all-shy groups	Pruitt and Keiser 2014
Anti-predator behavior	*S. dumicola*	During a predator attack, mixed colonies of bold and shy individuals show more defensive behavior than homogenous colonies	Wright et al. 2016
Efficient movement to resources	Sheep, *Ovis aries*	Compared with homogeneous herds, herds composed of both bold and shy sheep utilize more food patches over time while also maintaining cohesion	Michelena et al. 2010
	Forest tent caterpillar, *Malacosoma disstria*	Groups composed of both active and inactive caterpillars optimize cohesion and ability to collectively locate food	Dussutour et al. 2008
		*Theoretical model supports this particular result	Nicolis et al. 2008
	Cockroach, *Periplaneta americana*	Individuals in groups with variation among individuals in time spent sheltering move to shelters with a more optimal speed-accuracy tradeoff	Planas-Sitja et al. 2015
	Fish model	Variation in sociability of fish agents improves efficiency of movement to a target without losing cohesion	Couzin et al. 2005
	Robot swarm	Swarms are more likely to have successful initiations of group movements if there is a mix of “bold” and “shy” behavioral types	Eskridge and Schlupp 2014
Response to environmental variation	*A. studiosus*	Mixed groups of aggressive and docile spiders experience stable reproductive output (number of egg cases) at high and low temperatures while groups of all-aggressive or all-docile individuals experience lower reproductive output at high and low temperatures, respectively	Goulet et al. 2016
Increased Cooperation	Human model	Increased variation in male aggression leads to higher cooperation and reproductive output in “bully-victim” model	Gavrilets 2012
	Non-explicit animal model	High levels of cooperation become stable in a Prisoner’s Dilemma game if extrinsic factors maintain behavioral variation among the players	McNamara et al. 2004
Collective decision-making	Ant, *Temnothorax albipennis*, model	Simulated groups with a normal distribution of slow- to fast-assessing ants select nest-sites faster, and make more accurate decisions when only low-quality sites are available than homogenous simulated groups	O’Shea-Wheller et al. 2017
	Zebrafish, *Danio rerio*	Although not explicitly compared with homogenous groups, groups containing fish that consistently make fast-inaccurate decisions and those that make slow-accurate decisions make decisions with higher accuracy than either type of fish when assayed alone	Wang et al. 2015
Human team performance	*Homo sapiens*	Variation in extraversion of team members positively correlates with the team’s customer service and task performance ratings	Neuman et al. 1999
	*H. sapiens*	Teams of business students with greater variation in extraversion have higher oral presentation scores	Mohammed and Angell 2003

**Table 3:** Hypotheses and predictions for the effect of among-individual behavioral variation on group performance

Hypothesis	Predictions	Alternative Hypotheses	Predictions
H 1. Group performance is improved, rather than impeded, by among-individual variation in behavior	P 1. Measures of group success will increase with the repeatability of assayed behaviors (here, repeatability should be calculated at the group-level). Proxies of group success to be measured can include reproductive output, foraging efficiency, efficiency of collective movement, etc.	AH 1. Among-individual variation has no effect on group performance	P 1. Measures of group success will not change significantly with increasing repeatability of behavior
		AH 2. Among-individual variation within a group negatively impacts group performance due to the resulting increase in mismatches among individuals in their preferences and priorities	P 1. Measures of group success will decrease with increasing repeatability of behavior
		AH 3. Within-individual variation, rather than among-individual variation improves group performance	P 2. Groups with greater repeatability of behavior will experience delays in consensus, increased dissent, physical conflicts, fission events, and social parasites
		AH 4. Group success leads to an increase in among-individual variation in behavior, as opposed to the reverse relationship	P 1. Measures of group success will increase with increasing total behavioral variation in the group, but decrease with increasing repeatability of behavior P 1. Measures of group success will not be correlated with the repeatability of behaviors assayed prior to group formation, but will be correlated positively with repeatability when the behaviors are measured after group formation P 2. Time-series analysis will reveal that changes in metrics of group success precede changes in repeatability of behavior

Among-individual variation in behavior can correlate with several measures of group success. Groups with greater among-individual variation can have higher direct measures of group reproductive success than homogeneous groups (Modlmeier and Foitzik 2011;Pruitt and Riechert 2011; Modlmeier et al. 2012). They also can experience increased foraging success (Dyer et al. 2009a; Pruitt et al. 2012; Aplin et al. 2014;Pruitt and Keiser 2014) and more effective anti-predator behavior (Wright et al. 2016). Additionally, the efficiency of collective movement to resources can improve with increased among-individual variation in behavior (Couzin et al. 2005; Dussutour et al. 2008; Nicolis et al. 2008; Michelena et al. 2010; Eskridge and Schlupp 2014; Planas-Sitja et al. 2015). Fitness in groups with greater among-individual variation can also be more robust to variable environments than that of homogeneous groups (Goulet et al. 2016). Theoretical models show that increased among-individual variation leads to higher cooperation and fitness (Gavrilets 2012), thus reinforcing empirical results. Despite these findings, a potential mechanism by which social heterosis acts to increase the performance of diverse social groups when they do outcompete more homogeneous groups has evaded this line of research.

While support for the benefit of among-individual variation within a group appears strong, behavioral diversity is certainly not universally advantageous. In fact, among-individual variation in behavior can be quite detrimental to group performance when variation leads to social parasitism (Giraldeau and Caraco 2000) or decreased group cohesion (Krause and Ruxton 2002; Conradt and Roper 2005; Ward and Webster 2016). Moreover, past work suggests that diverse phenotypes within a group can produce conflict due to mismatches in priorities or preferences of groupmates (reviewed in Conradt and Roper 2005; Conradt 2012). As preferences become increasingly unaligned or mutually exclusive, group performance may become negatively correlated with among-individual variation.

When group performance does indeed correlate positively with among-individual variation, it is possible that hypotheses alternative to social heterosis could explain the correlation (Table 3). For instance, behavioral variation within a group, and not necessarily the among-individual component of this variation, could be beneficial to groups. Within-group variation could be achieved by high within-individual variation and relatively low among-individual variation. We remain skeptical that such a group could parallel a group with consistent among-individual variation due to the possible costs of activational plasticity and the benefits of predictability in groupmate behavior. However, future studies could test this hypothesis by comparing the success of groups that are matched in their level of group-level behavioral variation but differ in their level of among-individual variation. It is also possible that as a group becomes more successful, the pressure on individuals to conform behaviorally is reduced, such that group success causes an increase in among-individual variation, as opposed to the reverse relationship. While this possibility could also explain a correlation between among-individual behavioral variation and group performance, the majority of studies presented in Table 2 consisted of experiments in which a researcher created groups of individuals with behavioral types that were measured prior to group formation and then compared a group-level response variable after group formation. This experimental nature ensures that group behavioral type composition does indeed have some causal effect on group performance. Nonetheless, further work should investigate the potential role of this reverse direction of causality.

Although there are reasons to find the results presented in Table 2 surprising, this research does parallel the more extensive research on genetic diversity within eusocial insect colonies. Studies of genetic heterogeneity in eusocial colonies provide strong evidence that group productivity and stability benefit from increased genetic diversity within groups (Page et al. 1995; Liersch and Schmid-Hempel 1998; Jones et al. 2004; Mattila and Seeley 2007; Oldroyd and Fewell 2007). Among other hypothesized mechanisms (Shermen et al. 1988; reviewed in Page 2013), genetic diversity is thought to improve group performance by increasing the efficiency of division of labor systems in social insect groups (Mattila and Seeley 2007; Oldroyd and Fewell 2007). The result that genetic diversity improves group performance may not apply to non-eusocial animal groups, because cooperation and group success can be thwarted by reduced relatedness via within-group conflict (Kamel et al. 2010; Krupp et al. 2011). However, the parallel between research on among-individual behavioral variation and research on genetic diversity remains useful because the hypothesized mechanism by which genetic diversity leads to improved group performance—increased efficiency in task allocation—may be a shared mechanism that also explains why groups with greater among-individual behavioral variation can outperform homogeneous groups (Jandt et al. 2014; Jeanson and Weidenmüller 2014).

By switching the focus from genetic diversity to behavioral variation itself, which is the cumulative phenotypic result of several factors (e.g., genes, environment, development), personality research can contribute to our understanding of the direct role of among-individual variation in task-independent axes of behavior in generating efficient social organization. Given that animal personality is widespread across taxa (Gosling 2001; Bell et al. 2009), we propose that among-individual variation in task-independent behaviors could potentially play a role in connecting the evolution of sociality to task allocation and improved group performance in some systems, and thus, might contribute to the success of group-living animals.

## WHAT IS A TASK?

To broaden our understanding of task allocation, we employ a modified definition of what constitutes a task. For example, Jeanne (1988) defined tasks as behaviors performed to achieve some colony-level purpose, which implies that the tasks are only for the benefit of the group, and tends to limit the scope to eusocial animals. We suggest that a task is more profitably defined as any behavior that positively affects the fitness of conspecifics within a social group by providing a good or service to those conspecifics. By this definition, individual task performers in a group can occupy complementary roles that are not overtly cooperative. Overt cooperation is not necessary because the goods or services that an individual produces for the group can be a by-product of selfishly motivated actions (i.e., by-product mutualism; West-Eberhard 1975; Brown 1983). For example, extremely shy individuals that seek refuge at the first sign of predator presence provide useful information for nearby groupmates and may perform an unintentional vigilance task for others upon group formation (Gil and Hein 2017). It is our goal here not to claim that all social roles (e.g., competitive roles, cheaters) or behaviors should be thought of as tasks. Rather, by establishing a taxon-independent definition of a task, we hope to facilitate the discovery of ecologically relevant task allocation systems across animal societies.

By redefining tasks, we allow our framework to draw upon behaviors and behavioral roles that have yet to be studied from a task allocation perspective. Previously researched social roles in animal societies provide useful examples of the applicability of our definition. For example, “policers” in groups of pig-tailed macaques, *Macaca nemestrina*, are important for reducing conflict and maintaining social order (Flack et al. 2006). Because conflict resolution is likely beneficial for all individuals involved, this social role can be considered as much a task as more tangible and traditionally studied tasks, such as brood care or nest maintenance. We similarly suggest that leader–follower dynamics should also be considered a form of task allocation, as both “leading” and “following” tasks provide a service for groupmates (Anderson and Franks 2001; Michelena et al. 2010; Aplin et al. 2014). While leaders ensure efficient acquisition of resources, followers promote essential group cohesion (Couzin et al. 2005; Dyer et al. 2009b; Aplin et al. 2014).

In addition to more discrete divisions of labor such as leader vs. follower, tasks can consist of completing one small component of a larger group activity. The partitioning of tasks can substantially increase group efficiency and productivity, and it has been a well-studied aspect of task allocation in eusocial insects (Jeanne 1986; Seeley 1995). However, other animals also split work among groupmates for specific tasks such as foraging. Reports suggest that cooperative hunters, such as lions (*Panthera leo*), Harris’s hawks (*Parabuteo unicinctus*), and Aplomado falcons (*Falco femoralis*), may partition prey capture tasks and that they may also show consistency in their role across different hunts (Hector 1986; Bednarz 1988; Stander 1992). Animals can exhibit clear task partitioning in other contexts as well, such as when offspring care tasks are split among a pair or group of caretakers (Clutton-Brock 1991). It is important to note here that groups can allocate tasks within one broad task domain (e.g., foraging or parental care), without necessarily allocating all group activities.

## BEHAVIORAL SYNDROMES AS A POTENTIAL MECHANISM UNDERLYING TASK ALLOCATION (FIGURE 1; III)

Common to many models of task allocation is the idea that individuals can specialize on specific tasks (i.e., they show consistent individual differences in their task-related behaviors) which may improve their task performance and generate greater efficiency for the group (Oster and Wilson 1978; Robinson 1992; Wahl 2002). “Task participation” describes the full task repertoire of an individual over some relevant time frame. “Task specialization” has been defined broadly as bias for a particular task (Johnson 2002) but also more specifically as the performance of a task to the exclusion or limitation of other tasks (Robson and Traniello 2002). To frame task specialization using terminology common to animal personality research, we define specialization as relative consistency in an individual’s task participation over time in conjunction with among-individual variation in this task participation. “Task proficiency” refers to an individual’s ability or skill in performing a task relative to that of other individuals (Dornhaus 2008). In this section, we evaluate how task-independent behavioral types can influence developmental trajectories that ultimately guide individual choices in task participation through mechanisms previously established in eusocial insect research (Table 4).

**Table 4:** Examples of task syndromes

Task-Independent Behavioral Types	Specialized Task	Animal	Reference
Fast–slow exploration	Fast-exploring individuals lead groups to foraging patch while slow explorers prevent group dissolution	Great Tits, *Parus major*	Aplin et al. 2014
	Fast explorers defend nest territory while slow explorers provision young (biparental care)	Great Tits, *P. major*	Hollander et al. 2008
	Fast explorers defend the nest (cooperative breeding)	Superb fairy-wrens, *Malurus cyaneus*	van Asten et al. 2016
Exploratory– non-exploratory	Exploratory helpers defend territory while less exploratory helpers maintain territory (cooperative breeding)	Cichlid fish, *Neolamprologus pulcher*	Bergmüller and Taborsky 2007
	Exploratory individuals likely specialize on nest site selection	Argentine ants, *Linepithema humile*	Hui and Pinter-Wollman 2014
Aggressive–docile	Aggressive individuals defend the nest while docile individuals take care of brood	Ants, *Temnothorax longispinosis*	Modlmeier and Foitzik 2011; Modlmeier et al. 2012
High–low sensory perception	In increasing order of their response threshold to sucrose solution, 1-week-old workers become water, pollen, and nectar foragers 2–3 weeks later	Honey bees, *Apis mellifera*	Pankiw and Page 2000
High-low learning ability	Learning ability is correlated with specialization on pollen foraging	Honey bees, *Apis mellifera*	Latshaw and Smith 2005
Active–inactive, positively phototaxic–negatively phototaxic	Individuals that are more active and phototaxic perform foraging tasks while inactive individuals perform tasks inside the nest	Ants, *Myrmica rubra*	Pamminger et al. 2014
Aggressive–docile, exploratory– non-exploratory	Early in life, individuals that are docile and less exploratory perform more brood care than same-aged counterparts	Ants, *Leptothorax acervorum*	Kühbandner et al. 2014
Aggressive–docile, exploratory-non–exploratory, active–inactive	Aggressive, exploratory, active individuals contribute more to territory defense than docile, less exploratory, inactive individuals	Cichlids, *N. pulcher*	Le Vin et al. 2011
Aggressive–docile, bold–shy, active–inactive	Aggressive, bold, and active individuals patrolled, while docile, shy, inactive individuals foraged and cared for brood	Ants, *Myrmica rubra* and *M. ruginodis*	Chapman et al. 2011

Individuals in social groups often specialize in a task because of inherent adaptation (Seeley 1982; Trumbo and Robinson 1997; Dornhaus 2008), which usually refers to morphological or physiological differences among individuals that predispose them to perform specific tasks. Individuals are typically more responsive to and more proficient at these tasks for which they are inherently adapted (Wilson 1974; Pirk et al. 2004). However, inherent adaptations can also be behavioral, and pre-existing behavioral differences between individuals within a group may allow for task allocation to occur based on these task-independent behaviors, such that individuals with particular task-independent behavioral types come to specialize on particular tasks. In a social cichlid fish, *Neolamprologus pulcher*, for example, individuals that are more willing to explore a novel environment in an isolated test are more likely to defend a communal territory from an intruding conspecific, while less exploratory individuals are more likely to maintain the breeding shelter (Bermüller and Taborsky 2007). These behavioral predispositions that guide later task specialization are not constrained to be in axes of variation that are typically the focus of personality studies. For example, in artificially selected honey bee, *Apis mellifera*, colonies, individual response to sucrose concentration as a newly emerged worker predicts specialization in pollen or nectar foraging 2–3 weeks later in life (Page 2013). Although pre-existing inherent behavioral predispositions can be more cryptic than morphological ones, the task syndromes that they initiate could potentially be just as effective for distributing work within a group.

Prior experience also plays a role in allocating tasks and creating task specialists. Work with eusocial insects shows that individual experience with a task and task-related stimuli can make an individual more likely to respond to that task again (Theraulaz et al. 1998). Repeated experience can, therefore, create a feedback loop that underlies specialization. Among-individual variation in task-independent behavioral type could contribute to task specialization by affecting the rate at which individuals encounter different task-related stimuli in a spatially heterogeneous environment, mirroring the “foraging for work” concept in the eusocial literature (Franks and Tofts 1994;Tripet and Nonacs 2004; Mersch et al. 2013; Crall et al. 2018). Pamminger et al. (2014), for example, showed that activity level and sensitivity to light predict the spatial preferences of ant workers in the nest and thus contributes to the separation of workers into foragers and within-nest brood caretakers. Furthermore, the success of an individual in performing a task can affect the likelihood with which it will continue performing that task (Ravary et al. 2007). Self-reinforcement mechanisms associated with task experience are a well-established feature of existing models of division of labor (Theraulaz et al. 1998). Consequently, it is possible for both differences in inherent adaptations and experience-based mechanisms, both associated with an individual’s task-independent behavioral type, to lead to among-individual variation in task participation and thus a task syndrome.

An increase in task proficiency over time is a central benefit of having task specialists (Seeley 1982; Jeanne 1986; but see Dornhaus 2008), and increases in proficiency are commonly thought to occur due to skill acquisition (Dukas and Visscher 1994; Dukas 2018). However, the significance of skill acquisition in traditionally studied task allocation systems (i.e., those of eusocial insects) is contentious due to the short-life span and small brain size of insects. Whether this is a valid critique (see Chittka and Niven 2009), we propose that increased proficiency in task performance through skill acquisition is an important part of our framework for two reasons. First, one of the goals of our framework is to expand task allocation research to animal groups outside of eusocial insects, such as mammal and bird societies, in which skill acquisition and development over time may play a more prominent role in improving task proficiencies. Second, individuals with different task-independent behavioral types may vary in their cognitive capability and their propensity to use previous experience and/or social information to guide decision-making (Guillette et al. 2009;Kurvers et al. 2010; Cole et al. 2011; Sih and del Guidice 2012; Dougherty and Guillette 2018). Thus, among-individual variation in behaviors outside of task domains could affect the role task proficiency plays in task allocation by influencing which types of individuals and which types of tasks are most shaped by skill acquisition.

In Table 5, we put forth a set of hypotheses and predictions that will facilitate future tests of the occurrence and prevalence of task syndromes, as well as the impact of these task syndromes on task proficiency.

**Table 5:** Hypotheses and predictions for the effect of task-independent behavioral type on subsequent task participation choices

Hypotheses	Predictions	Alternative Hypotheses	Predictions
H 1. Task participation decisions are driven by task-independent behavioral type	P 1. Variation in task participation and specialization will be predicted by variation in task-independent behavioral types (by definition forming a task syndrome between task-dependent and task-independent behavioral tendencies)	AH 1-1. Task participation decisions are not related to task-independent behavioral type	P 1. Variation in task participation will not be predicted by variation in task-independent behavioral types
		AH 1-2. Behavioral type may covary with other traits, such as age or morphology. Task participation decisions are driven by these other traits, not behavioral type.	P 1. Variation in task participation might appear to be predicted by variation in task-independent behavioral types, but including age and morphology along with behavioral type as predictor variables in a multivariate statistical model of task participation will reveal that behavioral type actually explains little of the variance in task participation
		AH 1-3. Task participation influences task-independent behavioral type, as opposed to the reverse relationship	P 1. Task participation choices will not be correlated with behavioral type measured prior to task performance, but will be correlated with behavioral type measured after task performance P 2. Time-series analysis will reveal that repeatability in task participation precedes repeatability in task-independent behaviors
H 2. An individual’s proficiency in various tasks is influenced by its behavioral type	P 1. Variation in proficiency in a task will be predicted by variation in behavioral types even before individuals have gained any experience in performing the task; or: P 2. Variation in proficiency in a task will be predicted by variation in cumulative time spent performing the task, which will be predicted by variation in behavioral type	AH 2-1. Task-independent behavioral type may affect task participation, but neither behavioral type nor consistency in task participation affect proficiency	P 1. There will be no correlation between an individual’s behavioral type and its skill at performing each task either before or after they have had the opportunity to gain experience in task performance

## TASK SPECIALIZATION AND ALLOCATION IMPROVE GROUP PERFORMANCE (FIGURE 1; IV)

To date, research and theory on task allocation, specialization, and group success has almost exclusively focused on human and eusocial insect societies. The division of labor among humans is unique in that cooperation often occurs within groups of individuals that are not closely related, an exception to the kinship-based cooperative relationships that generally structure many other animal societies (Axelrod and Hamilton 1981; Boyd and Richerson 2005). Still, general ecological and economic principles apply well to understanding the success of human groups across space and time: more cooperative groups outcompete non-cooperative groups and groups of specialists are able to outcompete groups of generalists (Richerson 1977; Boyd and Richerson 2009). The field of sociology even has a specific term, “organic solidarity,” which describes the interdependence and unification of humans within a society resulting from a specialized system of division of labor (Durkheim 1947). In further support of the framework, modern human task specialization (i.e., job choice) is highly dependent on an individual’s behavioral tendencies outside of the workplace (Tom 1971; Holland 1997).

The division of labor between reproductives and workers and among workers themselves in eusocial insect societies is believed to be a primary cause of their ecological dominance and evolutionary success (Oster and Wilson 1978; Wilson 2001; Hölldobler and Wilson 2009). In a broad taxonomic sense, the sheer biomass of eusocial insects relative to their less social counterparts in sympatry is often the first line of evidence used to assert that their task allocation structures are an important component of their success. This biomass disparity also holds true between humans and other terrestrial vertebrates (Boyd and Richerson 2009). Beyond differences in biomass, strong correlations exist between the complexity of the division of labor system of a eusocial colony, the degree of individual specialization, and the group’s ecological success (Jeanson et al. 2007; Johnson and Linksvayer 2010; Jeanson and Weidenmüller 2014). The possibility that these factors shape the success of groups in other taxa seems probable, given the generality of the principles underlying ergonomic efficiency, but this possibility remains to be thoroughly explored (Table 6).

**Table 6:** Hypotheses and predictions for the effect of task allocation that results from task syndromes on group performance

Hypothesis	Predictions	Alternative Hypotheses	Predictions
H 1. Adaptive task allocation systems result from task syndromes	P 1. Measures of group success and individual fitness will be higher in groups that show task allocation based on task syndromes compared with groups that show no such task allocation P 2. Task allocation regimes based on task syndromes will persist and increase in frequency over ecological and evolutionary timescales	AH 1. Task syndromes are not adaptive because: (see following sub-hypotheses)	P 1. Measures of group success and individual fitness will **not** be higher in groups that show task allocation based on task syndromes compared with groups that show no such task allocation
		AH 1.1. - task syndromes produce task allocation systems that are vulnerable to cheaters	P 2. (continued from the above general prediction) The number of individuals who perform no task will increase as time since group formation increases
		AH 1.2. - task allocation based on task syndromes produces an efficient division of labor, but one that cannot flexibly meet the changing needs of a group	P 2. Measures of productivity in groups that exhibit task syndromes will often be higher than those of groups that do not exhibit task syndromes, but the success of groups with task syndromes will decrease significantly more than that of groups without task syndromes when the requirements of a group change P 3. There will be no significant change in the distribution of labor when different limitations are imposed on the group

## FLEXIBILITY IN TASK ALLOCATION

Task allocation refers to both stable differences in task performance (division of labor) and the process by which groups shift individuals to other tasks as demand changes (Gordon 2016). In contrast to the benefits of specialization previously discussed, group-level inflexibility is a potential disadvantage of highly specialized groups (Robinson 1992; Bonabeau et al. 1998; Gordon 2016). However, an analysis of task participation patterns in social insect societies by Kolmes (1986) suggested that many division of labor systems, even in colonies with caste systems and task specialists, are adapted more for flexibly responding to environmental perturbations than strictly for maximizing efficiency. Further studies of eusocial insect colonies also suggest that individual specialization and colony-level flexibility are not mutually exclusive (Robinson 1992; Johnson 2003).

Group-level flexibility could potentially be achieved by activational plasticity at the individual level. While specialization implies relative consistency in an individual’s task-related behaviors, task specialists can still show some variation around their mean task behavior. Indeed, even some morphologically specialized eusocial insects are capable of temporarily switching tasks when colony demand changes (Wilson 1984; Brown and Traniello 1998). Individual activational plasticity in task-related behaviors can likely allow for group-level flexibility in task allocation in non-eusocial animals as well. Variation in task participation around an individual’s main task is particularly likely when task participation choices are driven by task-independent behavioral type, given that there is often significant within-individual variation around the central task-independent tendencies that might ultimately guide the task participation (Bell et al. 2009).

Additional work on eusocial insects has suggested that colonies might further circumvent inflexibility by relying on individuals that are more specialized (i.e., show less activational plasticity) for increased productivity and individuals that are less specialized for increased flexibility (Robinson 1992; Charbonneau and Dornhaus 2015a; Charbonneau and Dornhaus 2015b; but see Johnson 2002). Task allocation regimes that result from task syndromes may exhibit a similar mechanism to balance productivity and flexibility. An individual’s mean task-independent behavior can often covary with the variance in this behavior (Dingemanse et al. 2010). For example, individuals that use safe habitat more and feed less in the presence of a predator are more behaviorally flexible and cooperative than their bold groupmates (Westerberg et al. 2004; Magnhagen and Staffan 2005; reviewed in Magnhagen 2012). These findings support results from coping style research that suggest that aggressive, bold, proactive individuals exhibit less within-individual variation than shy, docile, reactive individuals (Koolhaas et al. 1999; Koolhaas et al. 2006). If variation in task-related behaviors indeed correlates with that of task-independent behaviors, task syndromes may establish a system in which individuals with particular behavioral types are more extreme specialists with rigid task syndromes, while those with other behavioral types show more variation in their task participation choices.

Group-level flexibility in task allocation might also be achieved by developmental plasticity at the individual level. Within-individual variation in task participation discussed thus far in this section represents activational plasticity and does not imply a change in task specialization. Individuals specialize on a given task but show some variation in their task-related behaviors such that they may sometimes perform other tasks. However, it is possible that through developmental plasticity, an individual’s task specialization might actually change over time. This mechanism of flexibility provides a slower and more permanent response to a perturbation in task demands. Honey bees exhibit this developmental plasticity when the population of brood care workers in a colony becomes insufficient, with older honey bees regenerating their hypopharyngeal glands and reverting from foraging back to nursing (Winston 1987; Robinson et al. 1992). The extent to which individuals exhibiting task syndromes experience gradual changes in their central task-independent and task-related behavioral tendencies over time to meet changing group needs remains unknown but is worthy of investigation.

## OPERATIONALIZING TASK SYNDROMES

Task syndromes arise when central tendencies in task-independent behaviors influence developmental trajectories that eventually result in task specialization. The process by which task syndromes develop is, therefore, an example of developmental plasticity. Accordingly, we can conclude that a task syndrome exists when prior task-independent behaviors and current task participation correlate statistically. Empirical tests of the existence of task syndromes will, therefore, require measuring individuals’ antecedent task-independent behavioral type, assessing the tasks in which individuals later participate, and testing for a correlation between them. In practice, this involves taking several longitudinal measurements of relevant behaviors, monitoring subsequent task performance over time, and then modeling task participation as a function of task-independent behavior. But how long should one wait between measuring task-independent behaviors and task performance? Furthermore, developmental plasticity can cause an individual’s reaction norm, and thus their central tendencies in both task-independent and task-related behaviors, to change over time. So, how does one decide when to measure them so as to maximize the chance that they are stable enough to determine the structure of correlation between them?

We devote the remainder of this section to putting forth guidelines and benchmarks that will answer these questions in order to aid future empirical tests for the presence of task syndromes. One should ideally measure task-independent behaviors before they might initiate the developmental pathways that result in task specialization and then measure task participation after the developmental change is complete. For animals with relatively discrete life stages (e.g., animals with a larval stage and adult stage) and animals with periodic general behavioral stages (e.g., animals that hibernate, animals that mate seasonally), we can infer that developmental change is most likely to happen during the transition between stages. Thus, we might expect task-independent behavioral type in one stage to correlate with task specialization in the next stage (e.g., the level of foraging activity under a high risk of predation during non-birthing season predicts task specialization in parental care during the birthing season). The stage in which to measure task-related behavior and the stage in which to measure task-independent behaviors should be dictated by when individuals most actively partake in tasks. Of course, empirical evaluations will require some system-specific deviations from the scheme established here. For example, with species that hibernate, it might be best to measure task-independent behaviors in one active season and then task participation in the next active season in order to avoid behavioral assays of a hibernating animal.

For animals that do not have discrete life stages and do not exhibit periodic behavioral patterns, we suggest measuring task-independent behavioral type when individuals are not fully matured, either sexually or morphologically, and correlating this behavioral type with task specialization when individuals are adults. These potential carryover effects, both between discrete and more continuous life stages, provide a good opportunity to test for task syndromes because task-related behaviors can be more easily isolated from task-independent behaviors due to developmentally or seasonally specific performance of tasks in particular task domains.

## FEEDBACKS FROM GROUP SUCCESS

Despite the presentation of group performance as the culminating response variable of our framework, group performance itself has significant feedback effects on key components of the framework. In the following sections, we incorporate both proximate and ultimate consequences of differential group performance to contemplate not only how task allocation can emerge from sociality, but also how selective pressures on the performance of a group and its members produce more complex social orders.

### Feedbacks to individual fitness (Figure 1; V_a_) and among-individual task-independent behavioral variation within a group (Figure 1; V_b_)

The survival and reproductive success of animals in stable social groups is often critically dependent on the success of their group (Wilson 1975; Fewell 2003; Wilson and Wilson 2007; Boza and Számadó 2010; Crofoot and Wrangham 2010). The impact of group performance on individual fitness is itself important, but it could also have significant consequences, both proximate and ultimate, on the amount of among-individual task-independent behavioral variation within a group. A group’s performance could impact its composition of task-independent behavioral types within one generation in two ways: by altering the mean behavior of current group members or by impacting group membership (i.e., immigration and emigration). Farine et al. (2015) suggest that, over longer time periods, the mean behavior of individuals might change in order to achieve a more adaptive distribution of behavioral types in the group. Individuals could use either global information sampling (Johnson 2008) or proxies of group state based on individual state (Seeley 1995; Toth et al. 2005; Toth and Robinson 2005) to track group-level performance and then adjust their behavior accordingly. This change in behavioral type is a result of developmental plasticity, and, therefore, could only occur on longer timescales, but empirical evidence suggests that changes in individuals’ mean behavioral tendencies over time do indeed occur (Favati et al. 2015; Costa et al. 2019; Monestier and Bell 2020).

The ability of individuals to adaptively change their long-term behavior and central behavioral tendencies based explicitly on metrics of their group’s performance and behavioral composition is highly intriguing but needs much further investigation. It is possible that some animals are not able to accurately track group performance and are, therefore, unable to adaptively shift their behavior. A related issue may be that individuals can and do respond to depressions in group performance but a lack of coordination in behavioral shifts by individuals within the group delays or impedes the establishment of optimal task-independent behavioral type composition. Of course, behavioral types could also be insufficiently flexible, especially as individuals age (Stamps and Krishnan 2017) or in individuals with very extreme behavioral types, and this inflexibility would also impede groups from effectively achieving optimal behavioral type distribution via developmental plasticity.

Without necessitating a shift in the behavioral reaction norm of individuals over time, a group’s performance could also affect among-individual variation in behavior by impacting group membership. Work with slender-billed gulls, *Chroicocephalus genei*, shows that group membership can change in response to poor group performance (Francesiaz et al. 2017). Although Francesiaz et al. (2017) did not explicitly consider personality, previous research in other animals shows that both an individual’s behavioral type and the composition of behavioral types in the prospective group can influence group membership decisions (Cote et al. 2012; Harcourt et al. 2009b; Hellström et al. 2016). Furthermore, colobus monkeys, *Colobus vellerosus*, make group membership decisions by avoiding phenotypes (e.g., sex) similar to their own (Teichroeb et al. 2011). Whether animals might use behavioral type-based similarity avoidance in group membership decisions to ensure that they join groups with significant among-individual variation in behavior is largely unknown, and we, therefore, need empirical work in this area.

It will be important for future work to test the hypothesis that an individual’s group choice reflects a preference for avoiding similar behavioral types against opposing hypotheses that explain group membership decisions. Instead of joining a group based on its composition of behavioral types, individuals could be assessing more obvious qualities of individuals in the prospective group such as sex, age, or body size (McRobert and Bradner 1998; Griffiths and Magurran 1998; Hoare et al. 2000), or even simpler criteria such as group size (Cote et al. 2012). Empirical work has also demonstrated that individuals can preferentially join groups with individuals that are more similar to them thereby further homogenizing the group (Harcourt et al. 2009b). This evidence is contrary to the hypothesis that individuals join groups that will minimize their niche overlap, and so further evidence is needed to analyze if and when individuals join groups of individuals that are behaviorally different from them rather than similar to them.

A group’s performance can impact the amount of task-independent behavioral variation among group members on an evolutionary timescale through three different selective pressures: disruptive selection, negative frequency-dependent social selection, and group selection. Models show that slight differences between groupmates in central behavioral tendencies can result in disruptive selection on behavioral types within the group and selection for increased social responsiveness (Johnstone and Manica 2011; Wolf et al. 2011), which drives further increases in among-individual variation (Dall et al. 2004; Harcourt et al. 2009a; Wolf et al. 2011; Wolf and Weissing 2012). While disruptive selection enhances among-individual variation in behavior, negative-frequency dependence can maintain this diversity by conferring a fitness advantage upon individuals with an underrepresented behavioral type (social selection theory; Wolf et al. 1999; Farine et al. 2015).

In some species, an individual’s fitness is highly dependent upon group dynamics, and group selection can, therefore, maintain an optimal composition of behavioral types within groups (Wilson 1975; Wilson and Wilson 2007; Huguin et al. 2018). In water striders, for example, aggressive males experience higher reproductive success than docile males, but all individuals experience extremely low reproductive success when a group contains several highly aggressive males (Eldakar et al. 2009; Eldakar and Gallup 2011). Therefore, multi-level selection maintains among-individual variation in aggression in water striders despite a selective advantage of high aggression at the individual-level.

### Feedbacks to task allocation (Figure 1; V_c_)

Because an individual’s task-related behaviors are part of its behavioral type, a group’s performance can impact its task allocation in much the same way it influences the distribution of the task-independent behavioral tendencies in the group. Poor group performance can lead to the reallocation of tasks (Mooney et al. 2015). Task switching has received significant attention in the eusocial literature (Gordon 1989, 1996; Johnson 2002), but Biro et al. (2016) suggest that there is a time-depth component of collective behavior in both eusocial and non-eusocial groups, by which animal groups might evaluate metrics of previous performance and reallocate social roles accordingly. Due to a correlation between behavioral tendencies unrelated to tasks and social role (Montiglio et al. 2013), this task reallocation likely occurs simultaneously with the proximate and ultimate changes in task-independent behavioral type distribution that occur by the mechanisms established above. If task-related behavioral tendencies indeed form a task syndrome with task-independent behavioral tendencies, then efficient allocation of tasks could also evolve with the evolution of an optimum composition of task-independent behavioral types. Thus, although primitive task allocation that results from task syndromes could potentially arise shortly after group formation, it could evolve and be enhanced into more nuanced task allocation systems seen in some taxa today.

### Feedbacks to sociality (Figure 1; V_d_)

Improved group performance as a result of task allocation also influences the evolution of sociality. The evolution of grouping depends critically on the costs and benefits of group-living (Krause and Ruxton 2002). Once sociality is established, however, increasing the benefits of sociality by the mechanisms presented in this framework (i.e., higher productivity and efficiency due to task allocation) could render the evolutionary transition from social- to solitary-living less likely in some cases. Theoretical models predict a positive feedback loop of sociality, in which the interdependence of groupmates, which could result from task allocation, leads to increasing interdependence (El Mouden et al. 2010; Lehtonen and Kokko 2012). The most extreme form of sociality—eusociality—is generally accepted as an evolutionary endpoint from which a species cannot return (Foster 2009; Hölldobler and Wilson 2009), and the positive feedback in our framework (sociality ↔ personality diversification ↔ group success ↔ sociality) hints that all social species might resist the tendency to revert back to solitary-living because of socially selected task allocation patterns. A phylogenetic analysis of primates confirms that while the evolutionary transition from group- to solitary-living is possible, it is rare (Shultz et al. 2011).

Alternatively, it is possible that the occurrence of task syndromes as a result of the framework that we present can actually make a group vulnerable to social parasites. If individuals with certain task-independent behavioral tendencies consistently perform the most relevant tasks for the group, it is possible that behavioral tendencies of other individuals in the group do not predispose them to perform a particular task, or even predispose them to perform no task. This situation might occur with individuals that vary in their general activity level, with the most active individuals performing several tasks and the least active individuals performing no task. The least active individuals in this case will benefit from the tasks performed by their more active groupmates, while providing no benefit to these groupmates. This social parasitism could reduce the benefits of group-living, thus increasing the likelihood of an evolutionary reversal to solitary-living.

## CONCLUSION

Despite decades of research into the mechanisms of caste-based task allocation in eusocial insects, studies on mechanisms of task allocation that do not depend on age- or morphologically-based castes remain comparatively scant. In evaluating the literature, we found that among-individual variation in central behavioral tendencies unrelated to tasks could provide a sufficient mechanism for task allocation in species that do not have discrete castes, and that this task allocation might explain the interesting finding that has emerged in several taxa that animal groups containing greater among-individual variation in behavior can be more successful than more homogeneous groups. This extensive review of the literature lends credibility to the field of study at the intersection of social behavior and animal personality.

As a critical next step, we suggest future research test the predictions developed herein to evaluate the applicability of the framework to diverse taxa as well as the potential for alternative hypotheses (summarized in Tables 1, 3, 35, 36). In this review, we have suggested that any social group might be capable of exhibiting task syndromes, but more research is needed to more fully understand under what circumstances one or more components of the framework break down, thus breaking the link between sociality and task allocation. Species with facultative task allocation, in which some groups allocate tasks while others do not, could serve as ideal systems in which to test alternative hypotheses. Additionally, groupmate interactions can range from cooperative to hostile. We have focused herein on cases of cooperative, or at least complementary, roles, but if early systems of task allocation that result from task syndromes generate conflict and cheating, then this could undermine the evolution of adaptive task allocation structures. To inform the generalizability of our framework, future research, both theoretical and empirical, should investigate the extent to which groupmates in conflict-based roles are capable of adaptive task allocation.

We urge empiricists to consider the possibility that some groupmates that seem to be in conflict, may actually occupy complementary roles and exhibit task allocation. For instance, scroungers in groups with a producer-scrounger dichotomy are considered parasites on their producer groupmates. However, both Dyer et al. (2009a) and Kurvers et al. (2009b) found that boldness predicts producer–scrounger tactics, and they suggest that bold producers may actually benefit from the caution and vigilance of shy scroungers. Therefore, in some cases, social host–parasite relationships may be a misrepresentation of underlying mutually beneficial task syndromes.

As a final point, this framework has implications beyond behavioral ecology. As Krause et al. predicted in 2010, the rise of animal personality research has drawn interesting parallels to our own species. Studies show that personality diversity in human groups is positively correlated with group performance (Neuman et al. 1999; Mohammed and Angell 2003). Our review draws findings from across the field of animal behavior to support the conclusion that diverse groups can be more productive than homogenous groups, and we provide an explanation—task allocation arising from among-individual variation in task-independent behavioral tendencies—for this pattern that is thoroughly applicable to human groups as well.

## Data Availability

This article has no additional data.
